# XX Male: Early Detection With Prenatal Testing

**DOI:** 10.7759/cureus.48946

**Published:** 2023-11-17

**Authors:** Ayah Ibrahim, Jordyn Mullins, Scott Cyrus

**Affiliations:** 1 Pediatrics, Burrell College of Osteopathic Medicine, Las Cruces, USA

**Keywords:** noninvasive prenatal test, level 2 ultrasound, klinefelter syndrome, fluorescence in situ hybridization (fish), genetic karyotype, de la chapelle syndrome, sry gene, disorder of sex development (dsd)

## Abstract

A 46,XX male represents a variant of Klinefelter syndrome (47,XXY), under the category of a disorder of sex development (DSD). Despite possessing an XX karyotype, these individuals exhibit a male phenotype, which, in this case, results from a translocation of the SRY gene from the Y chromosome onto the X chromosome. This genetic alteration results in the development of male gonadal characteristics. This case report outlines a prenatal diagnosis of a 46,XX female in conflict with a level 2 ultrasound. It details the patient's presentation, diagnosis of an SRY-positive 46,XX male, and medical history. The discussion focuses on the advantages of early identification and intervention in managing symptom progression and addressing fertility challenges through hormone replacement therapy. Further exploration of 46,XX DSD early detection and the underlying mechanisms is essential for refining diagnostic and therapeutic approaches that result in a greater quality of life for these patients.

## Introduction

46,XX disorder of sex development (DSD), a variant of Klinefelter syndrome (47,XXY) (KS), also known as de la Chapelle syndrome, affects approximately one in 20,000 newborn males annually [1]. Most newborn males with this condition exhibit a typical male phenotype, making a prepubertal diagnosis uncommon due to a lack of signs and symptoms. Studies suggest that individuals present with a tall and narrow frame, broad hips, gynecomastia, hypogonadism, reduced testicular function, lack of general male secondary sex characteristics, and potentially reduced verbal intelligence. Most of these symptoms will manifest during or after puberty [2].

Sex determination during fetal development is influenced by the presence or absence of the sex-determining region Y (SRY) gene located on the Y chromosome inherited from the paternal spermatocyte. The SRY gene, located on the short arm of the Y chromosome, plays an absolute role in differentiating the undifferentiated gonads into the testes and supporting the formation of phenotypic male genitalia. However, in cases such as 46,XX DSD males, individuals do not possess an entire Y chromosome yet exhibit a male phenotype. Several mechanisms have been suggested to elucidate the origins of XX sex reversal. Among these, one involves the translocation of a portion of the Y chromosome, encompassing the SRY gene, to either an X chromosome or an autosome. Another mechanism within the testis-determining pathway arises from a mutation in an unidentified X-linked or autosomal gene. This mutation triggers gonadal differentiation into the testis, even in individuals without the SRY gene. Lastly, within gonadal tissue, the presence of a concealed Y chromosome mosaicism has also been considered [3].

This case report presents the findings of a now 2-year-old who was identified as a SRY-positive 46,XX male and reviews the current literature regarding potential treatment options and limitations to treatment. The discovery was made by noting a discrepancy between two noninvasive prenatal tests (NIPT) and a fetal level 2 ultrasound anatomy scan. Upon delivery, the patient exhibited palpable testes in the scrotum and underwent circumcision.

This case report was presented as an abstract at the New Mexico Osteopathic Medical Association 43rd Balloon Fiesta Medical Symposium on October 13, 2023.

## Case presentation

**Figure 1:**
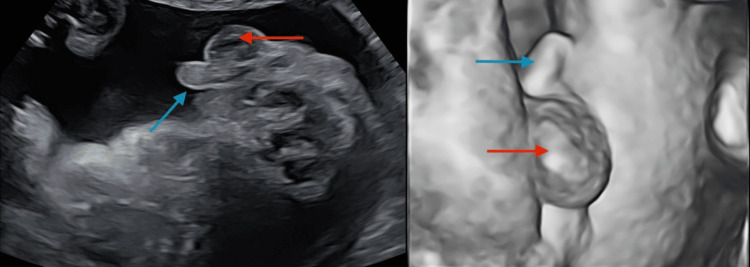
The mother's 20-week level 2 ultrasound scan showing the presence of male genitalia. In both photos, the red arrow indicates the scrotum and the blue arrow indicates the penis.

A 23-year-old Caucasian mother, pregnant with her fourth child all from the same biological father (gravida 4, para 3, stillbirth 0, spontaneous abortion 1, live birth 3), underwent an initial personal preference single nucleotide polymorphism NIPT by 10 weeks' gestation to discover the baby's sex. The initial NIPT results indicated no presence of Y chromosome DNA, suggesting she was carrying a female fetus. The accuracy of the current NIPT was felt to be reliable, as she had used similar elective testing to discover the sex of her two older sons, both showing positive DNA for the Y chromosome. However, a 20-week anatomy scan, also known as a level 2 ultrasound, revealed the presence of male genitalia, contradicting the earlier NIPT findings (Figure 1). A subsequent NIPT was conducted to address this discrepancy, yielding identical results with no presence of Y chromosome DNA. After the conclusive photographic evidence from the level 2 ultrasound showing male genitalia, the family was resolved to the fact that the NIPT results were in error. Therefore, no further genetic counseling or invasive genetic testing was deemed necessary or desired. The phenotypic male infant was delivered at 39 weeks' gestation via spontaneous vaginal delivery, exhibiting gestational age-appropriate male genitalia with palpable testes in the scrotum and congenital phimosis of the penis. The infant's body weight at birth was 7 lbs 4.8 oz (3310 grams), length was 19.49 inches (49.5 cm), and head circumference was 13.58 inches (34.50 cm). The infant underwent circumcision on the second day of life, as requested by the parents, and met all the discharge parameters. The infant was seen in the pediatrician's office at five days of age.

**Figure 2:**
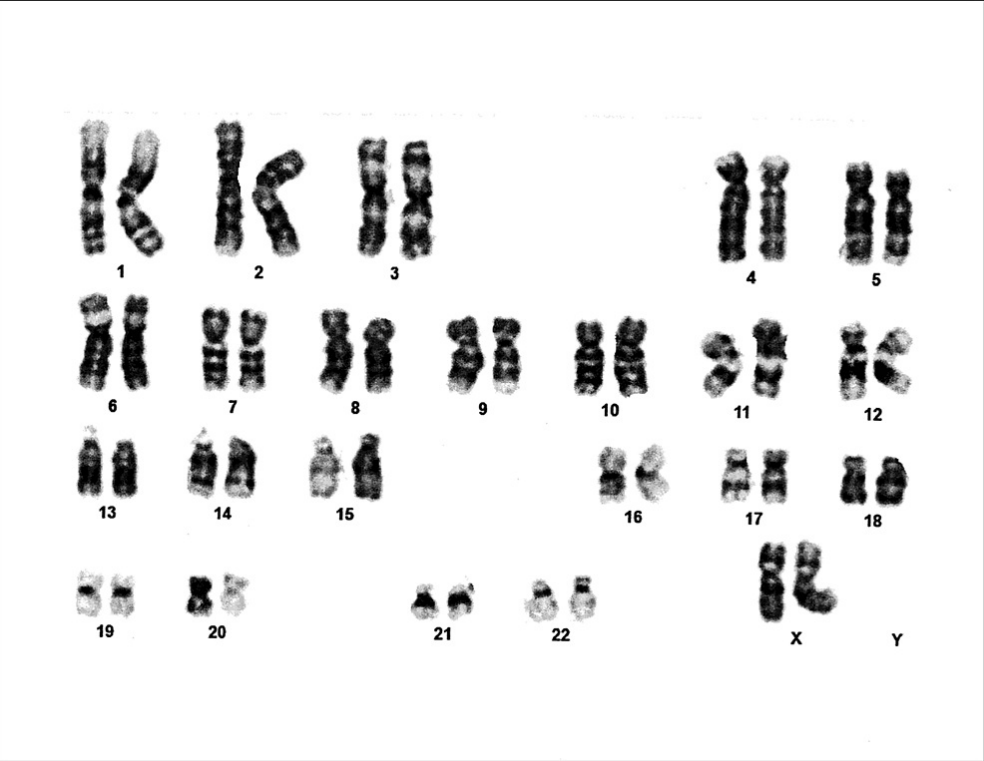
Karyotype analysis revealing two X chromosomes and the absence of a Y chromosome.

**Figure 3:**
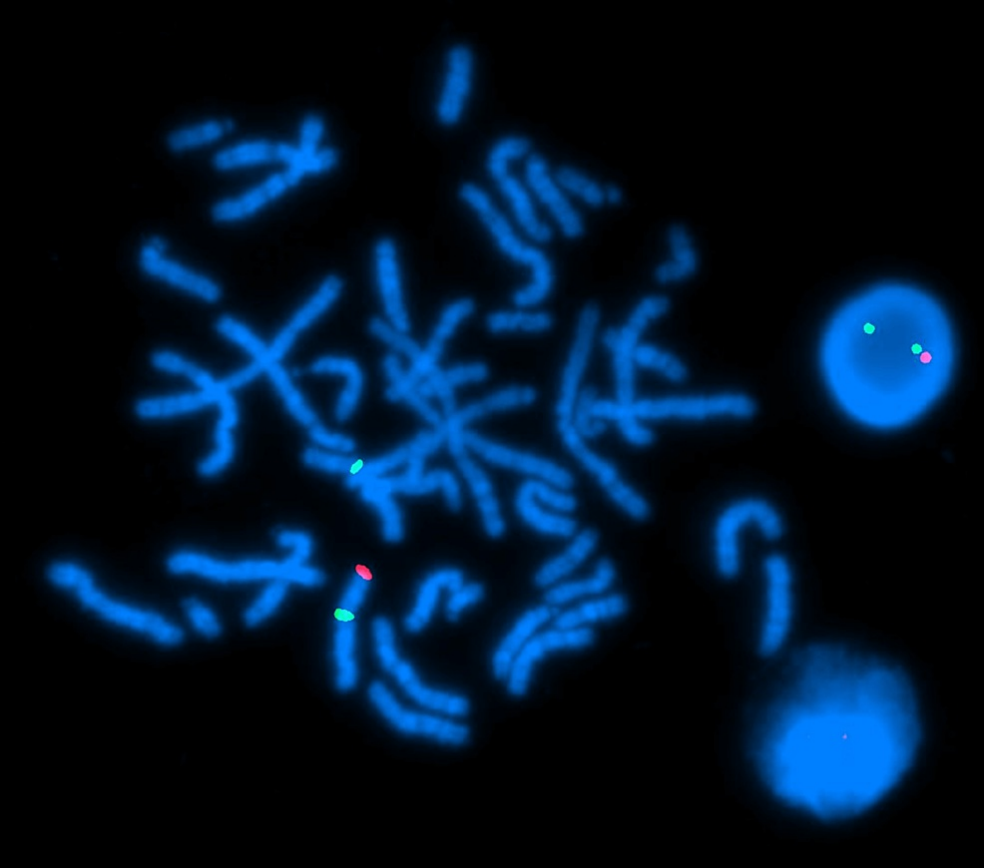
A FISH of metaphase chromosomes, shown in blue. The FISH displayed two X chromosomes (green regions) and the presence of the SRY gene (pink region). FISH: fluorescence in situ hybridization

Cord blood was collected at delivery for genetic karyotyping, because of the inconsistent results with the previous two NIPT and the current phenotypic male appearance of the infant at delivery. Karyotype analysis resulted in 46,XX female (Figure 2). After a discussion between the physician and the genetics lab, further in-depth genetic analysis was mandatory. A sample of cord blood underwent fluorescence in situ hybridization (FISH) studies for the X and Y chromosomes. The FISH studies probed for the X chromosome centromere revealed a hybridization pattern of two X chromosomes. Additionally, the FISH studies with the SRY probe specific to the Y chromosome showed the presence of the SRY gene on the distal end of the short arm of one of the X chromosomes (Figure 3). These results indicated a translocation with breakpoints located in the distal Xp and Yp pseudoautosomal regions, resulting in an active SRY gene on one of the X chromosomes, promoting a male phenotype. 

At approximately five weeks of age, an ultrasound of the infant's scrotal, pelvic, and abdominal anatomy revealed both testes to be normal size and echogenicity with a small left hydrocele. The bladder, liver, spleen, and bilateral kidneys showed normal appearance. Of note, there was the absence of ovaries, fallopian tubes, and uterus. Laboratory testing for luteinizing hormone (LH), follicle-stimulating hormone (FSH), and testosterone were not obtained. After consultation with the parents, it was determined that raising the infant as a male would be most appropriate. Endocrinology was consulted to oversee appropriate future hormonal treatment.

Over the course of the child's first two years, appropriate developmental milestones were maintained. The child stayed within the normal range (fifth to 85th percentile) for weight, length, and head circumference. At the child's two-year well-child check, weight was in the sixth percentile, and height was in the eighth percentile on the male Centers for Disease Control and Prevention (CDC) growth chart.

## Discussion

Translocation of the SRY gene from the Y chromosome to the X chromosome has been documented previously in the literature, with the first FISH displayed in 1992 [4,5]. During gametogenesis, gametes will undergo two rounds of meiosis. One complication that can occur during this process is translocation of genetic material from one sex chromosome to the other, likely during paternal meiosis. In this case, the genetic material which translocated from the Yp chromosome to the Xp chromosome involved the SRY gene. The SRY gene is then expressible, resulting in the production of anti-Mullerian hormone (AMH), which is responsible for the degeneration of the Mullerian ducts and promoting the development of the Wolffian ducts, for female and male gonadal development, respectively. Therefore, a 46,XX genotype can result in a male phenotype due to the promotion of Wolffian duct development from SRY expression on the X chromosome, which is elucidated elsewhere [6]. The main complication is testicular dysfunction resulting in hypergonadotropic hypogonadism. This is due to non-functional gonads, with the only source of steroidogenesis occurring in the adrenal glands and peripheral adipose tissue [6]. Associated with this diagnosis is azoospermia, which has been well documented [7,8]. Due to this, these patients will often require hormone replacement therapy (HRT) to achieve secondary sex characteristics, and to conceive offspring, they will require assisted reproductive technology (ART) or seek adoption [7].

Research supports that early detection and diagnosis can assist in dampening the typical signs and symptoms associated with 46,XX males [7-9]. Classically, treatment has been initiated in postpubertal development due to symptomology not appearing until these years. Some of these symptoms, such as gynecomastia, may be permanent. Therefore, it would be advantageous to begin HRT prior to pubertal years, when there is typically a rise in testosterone in developing males. In doing so, it may be possible to prevent, or at least blunt, the development of gynecomastia and promote the development of secondary male characteristics [9]. Limited research exists specifically for 46,XX patients. Spermatogenesis relies on several genetic loci on the Y chromosome, including but not limited to USPY9 and UTY [10]. If these gene loci did not translocate to the Y chromosome in a 46,XX patient, then these patients are most probably infertile from birth, and intervention would not be beneficial. 

Early detection may also be important in order to rule out the possibility of developing certain malignancies, such as gonadoblastoma. These tumors are well known to arise in undescended testicles, commonly seen in syndromes such as KS and 46,XX DSD [11]. This tumor is highly correlated with the GBY gene on the Y chromosome [11]. Therefore, in a 46,XX DSD male, it would be necessary for that gene to translocate as well, which is unlikely. However, it may be appropriate to screen for in order to respond appropriately with treatment, such as an orchiectomy, at a younger age to avoid metastasis, which is commonly seen in KS [11].

Recent research indicates a growing belief that fertility preservation can be achieved in KS patients through sperm retrieval [12]. Infertility in KS patients stems from progressive testicular atrophy during puberty. Prior to puberty, these patients exhibit testosterone, LH, and FSH levels within the normal levels for a male. However, as they approach puberty, testosterone levels decrease, while LH and FSH levels rise. Consequently, the retrieval and cryopreservation of sperm before puberty or during adolescence are showing promising prospects for future fertilization [12]. KS patients have a functioning Y chromosome; hence, sperm retrieval and cryopreservation show promise. For 46,XX patients, sperm retrieval may be possible if the patient has a translocation of the necessary gene loci required for spermatogenesis [10]. This novel research holds promise for future families seeking fertility solutions.

Traditionally, HRT has been the recommended course of action due to the declining testosterone levels and increasing LH and FSH levels during puberty. This recommendation originates from the malfunctioning Leydig cells in KS patients, impairing their ability to produce testosterone even when stimulated by elevated LH levels. Notably, recent studies have indicated that Leydig cells can respond to human chorionic gonadotropin (hCG), leading to testosterone production. This discovery has expanded the potential treatment options for KS patients [13].

Beyond the ongoing research on HRT and its potential advantages for enhancing the quality of life and fertility among 46,XX patients, thought-provoking questions emerge concerning the societal dimensions of this medical condition. Although 46,XX DSD males could mistakenly be designated as intersex individuals, this genetic condition should be in conflict with matters such as gender identity, the upbringing of children according to their assigned sex at birth, and their involvement in gender-specific sports. These unique cases prompt further contemplation and exploration of state and federal legislative matters that address the medical use of HRT.

## Conclusions

This report sheds light on a case of an SRY-positive 46,XX male DSD, emphasizing the importance of thorough genetic testing and clinical assessment to accurately diagnose such cases. The translocation of the SRY gene from the Y chromosome to an X chromosome, resulting in a male phenotype, challenges traditional concepts of sex determination. Early detection and intervention hold significant potential to mitigate the development of certain symptoms, such as gynecomastia, and to address fertility issues through HRT and ART. Further research into the molecular mechanisms underlying these conditions and their implications will aid in refining diagnostic approaches and optimizing therapeutic strategies for affected individuals. 
